# Emotional Development in Adults with Autism and Intellectual Disabilities: A Retrospective, Clinical Analysis

**DOI:** 10.1371/journal.pone.0074036

**Published:** 2013-09-16

**Authors:** Tanja Sappok, Jan Budczies, Sven Bölte, Isabel Dziobek, Anton Dosen, Albert Diefenbacher

**Affiliations:** 1 Evangelisches Krankenhaus Königin-Elisabeth-Herzberge, Department of Psychiatry, Berlin, Germany; 2 Charité, Institute of Pathology, Berlin, Germany; 3 Department of Women’s and Children’s Health, Center of Neurodevelopmental Disorders at Karolinska Institutet (KIND), Stockholm, Sweden; 4 Cluster of Excellence Languages of Emotion, Freie Universität Berlin, Berlin, Germany; 5 Department of Psychiatry, University Hospital, Radboud University, Nijmegen, The Netherlands; University of Tuebingen Medical School, Germany

## Abstract

Individuals with intellectual disability (ID) are at risk for additional autism spectrum disorders (ASD). A large amount of research reveals deficits in emotion-related processes that are relevant to social cognition in ASD. However, studies on the structure and level of emotional development (ED) assessing emotional maturity according to the normative trajectory in typically developing children are scares. The level of ED can be evaluated by the ‘Scheme of Appraisal of Emotional Development’ (SAED), a semi-structured interview with a close caregiver. The SAED assesses the level of emotional developmental based on a five stage system in 10 domains, for example, ‘interaction with peers’ or ‘object permanence’, which are conducive to the overall emotional developmental level. This study examined the ED as measured by the SAED in 289 adults (mean age: 36 years) with ID with and without additional ASD. A lower level in ED was observed in ASD/ID combined that corresponded to the ED of typically developing children aged 1.5–3 years versus an ED with a corresponding age of 3–7 years in ID individuals without ASD. Moreover, distinct strengths in ‘object permanence’, and weaknesses in ‘interaction’, ‘verbal communication’, ‘experience of self’, ‘affect differentiation’, ‘anxiety’, and ‘handling of material objects’ led to a characteristic pattern of ED in ASD. SAED domains with highest discriminative power between ID individuals with and without ASD (5/10) were used to predict ASD group membership. The classification using a selection of SAED domains revealed a sensitivity of 77.5% and a specificity of 76.4%. ASD risk increased 2.7-fold with every SAED level. The recognition of delayed and uneven pattern of ED contributes to our understanding of the emotion-related impairments in adults with ID and ASD these individuals. Assessment of intra-individual ED could add value to the standard diagnostic procedures in ID, a population at risk for underdiagnosed ASD.

## Introduction

Individuals with intellectual disability (ID) have an increased risk for autism spectrum disorders (ASD) compared to the general population [1]–[3]. ASD, however, often remains unrecognized in ID individuals [4], [5]. Because of symptomatic overlaps between ID and ASD, and the lack of standardized appropriate measures, diagnosing ASD in adults with ID remains challenging [6]. Currently, the ‘Scale for Pervasive Developmental Disorders in Mentally Retarded Persons’ (PDD-MRS) and the ‘Autism Spectrum Disorders - Diagnosis for Adults’ (ASD-DA) [7], [8] are the only ASD measures that are adapted for ID. However, they are screeners, only generating suspicion of ASD, unable to confirm an ASD diagnosis. Individuals who are affected by both ASD and ID belong to the most pharmacologically treated mental health population. They show low outcomes and pose high demands to caregivers [9]–[12]. Therefore, identifying additional ASD in ID individuals is warranted to improve mental health and the quality of life.

According to DSM-IV-TR and ICD-10 criteria, ASD is a pervasive developmental disorder that is defined by early onset impairments in social reciprocity alongside with restricted, repetitive behaviors [13], [14]. Cognitive concepts such as executive malfunctions [15], weak central coherence [16], and problems with ‘theory of mind’ [17], [18], attempt to explain the basis of autistic symptomatology. Other psychological theories propose emotion-processing alterations as the core ASD deficits. For example, Hobson and others describe difficulties in perception, recognition, understanding, expression, and regulation of emotions [19]–[23]. In fact, Leo Kanner (1943) himself first delineated alterations in the emotion system, which he summarized as a lack of “affective contact” [24]. The emotion and the social cognition system are closely related networks [25], [26]. Impairments in social reciprocity, such as lack of joint attention, reactive smiling, imitation, and eye gaze processing, are precursors of ‘theory of mind’ deficits observed in ASD [27], [28], and reduce opportunities to share emotional experiences with others [28]. These missed opportunities result in reduced social competencies and impoverished maturation of socio-emotional abilities. Cognitive theories hence might not be sufficient to fully account for the psychological nature of ASD [29], [30].

Comprehensive evaluation of the *development* of the emotion system has received limited attention so far in ASD. The evolution of emotional maturity follows a progressive sequence of qualitative changes [31]–[33]. Newborns are already emotionally capable individuals who show, perceive, and respond to a range of simple emotions [30], [32], [34], [35]. Within the first year of life, the emotional response is modulated by the behaviors of the interaction partners [34]. In the second year of life, experiences of ‘joint attention’ with the caregiver toward an object evolve joyful affects [36], [37]. Gradually, emotional responses and regulation become more complex, e.g., manipulating the emotional states of others by teasing or cooperating in approximately the third year of life [38]. Preschoolers can increasingly regulate their affective states and understand the causes and consequences of emotions, with further progress in empathy and pro-social behaviors in school-aged children [39]. Age-appropriate changes in the emotion system are the basis for the onset of self-concept and the formation of personality structures [40]. These developmental achievements of emotional competence can be assumed to be the products of various internal and external factors. First, basic brain systems that are responsible for survival, such as feeding, protection, reproduction, or social contact, are conducive for basic emotions and result in motivations that are responsible for a specific behavior, e.g., searching for food [41]. Second, emotional development (ED) is stimulated and closely accompanied by cognitive developmental changes [33], i.e., object permanence. For secure attachment, the development of an inner representation of the outside environment as revealed by ‘object permanence’ is a fundamental prerequisite. Third, ED is closely connected to social interaction processes: Caretakers mirroring the child’s internal emotional states in a setting of a secure early attachment relationship trigger and support further development of ED [31], [42], [43]. Finally, the situational context must be appreciated because it could alter the evaluation of emotional reactivity, and it could influence emotion regulation strategies [44], [45]. In short, the course of ED is inevitably intertwined with social interaction, perceptual and motor functions, physiological reactivity, and certain cognitive abilities. Albeit possibly delayed and incomplete, individuals with ID principally pass the same periods of ED as normally developing children do [46]–[49].

**Figure 1:**
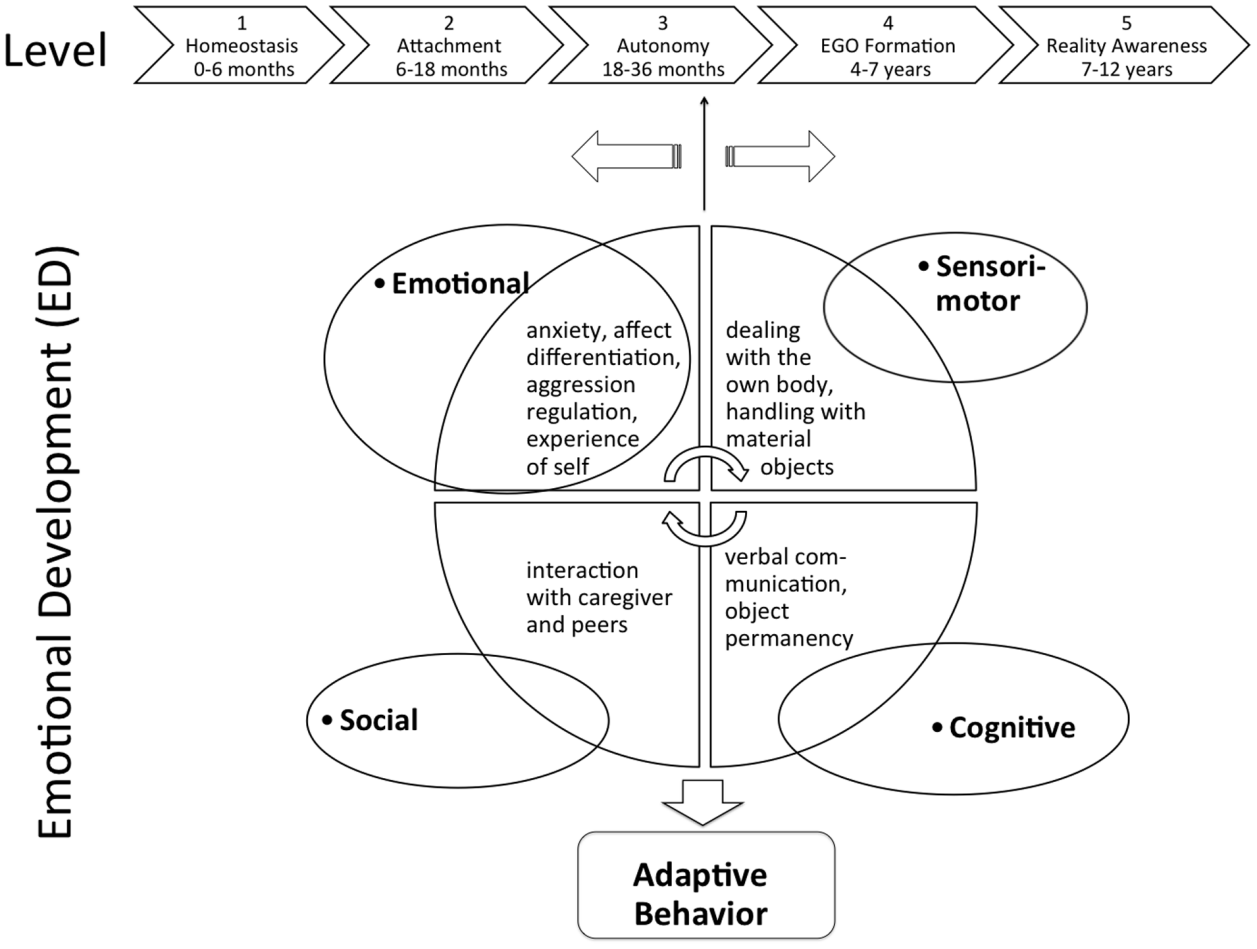
Conceptualization of the various components of ED, its dynamic aspects and its behavioral impact. Different aspect of personality structure, i.e., emotional, sensorimotor, communicative, and social undergo certain developmental steps ( = levels) throughout infancy. Each level of ED is associated with a distinct range of adaptive behavior.

Here, we consider the ‘concept of ED’ in terms of the ‘developmental approach’ outlined above, i.e., emotional maturity according to the normative trajectory in typically developing children. Consequently, the ‘concept of ED’ incorporates predominantly emotional but also social, sensorimotor, and cognitive functions on the background of normal development in infants [50]–[53]. The various components of ED reciprocally interact and stimulate each other for a further maturation of the individual and an optimal adaptation to the environment [54], [55]. This ability to adapt to everyday life is pivotal for living up to the personal and professional potentials of the individual and leading a content life. Figure 1 depicts these aspects integrated in the overall level of ED.

There is a substantial overlap of certain aspects of the ED with other theories that are in line with the developmental approach, e.g., social development as assessed by the ‘Socialization Area’ of the Vineland Adaptive Behavior Scale or the Children’s Social Behaviour Questionnaire [56], [57], sensorimotor functions as measured by the ordinal scales of Uzgiris-Hunt [58], or cognitive abilities according to Piaget [33]. As shown in Figure 1, these concepts of social, sensorimotor, and cognitive development are expanded by the emotional aspects to produce the overall ED. The developmental approach is elucidating the interplay among these aspects across the individual’s life span. Depending on the level of ED, certain capacities in self-regulation, behavioral control, and executive functions can emerge [59]. Therefore, the level of ED results in a distinct pattern of *adaptive or maladaptive behavior* that proceeds along a developmental trajectory (cf. Figure 1) [60].

A variety of scales aim to measure ‘emotional competence/intelligence’, an approach that is focused on emotional abilities, e.g., the appraisal and regulation of emotions; these scales are designed to assess performance similar to IQ tests [61], [62]. Bridging the gap between social neuroscience and developmental psychology, Dosen designed the ‘Scheme of Appraisal of Emotional Development’ (SAED) to assess emotional maturity according to the normative trajectory in typically developing children [40], [63]. The SAED is a semi-structured interview that is conducted by a significant other that classifies the current developmental level into 10 domains (cf. methods section) and provides an overall assessment of the individuals’ level of ED. Table 1 summarizes the different levels of ED, the age-equivalents of typically developing children, and the major achievements within a certain period.

**Table 1:** Levels of ED, age equivalents, and major developmental achievements.

Level of ED	Age	Developmental achievements
1: Adaptation	0–6 months	Homeostasis; integration of internal; e.g., sensory stimuli; bowel movements; and external stimuli; e.g., time; place; caregivers
2: Socialization	6–18 months	Attachment; emotional security; confidence
3: Individuation	18–36 months	Differentiation of self from others; autonomy; manage separation from bonding person; object permanence
4: Identification	3–7 years	Ego-formation; ego-centered; identification with important others; little abstract thinking; evolving social skills; learn by experience
5: Reality awareness	7–12 years	Reflective thinking; emerging moral thinking and self-respect; ego-differentiation; reasoning

Each level of ED is associated with specific basic emotional needs and motivations, different coping abilities, and consequently different behavioral patterns (cf. Figure 1). Consequently, the scheme shows a strong positive correlation with the Vineland Adaptive Behavior Scales, which is one of the most widely used tools used to assess adaptive abilities [64]. This study also suggested good reliability and validity of the measure.

Functions that are assessed by the SAED are prerequisites for social communication and interaction; these capabilities are impaired in individuals on the autism spectrum. For example, the domain ‘experience of self’ is conceptually closely related to self-awareness and therefore to ‘theory of mind’ abilities, which are known to be core problems in ASD [27], [32]. Moreover, the domains ‘interaction with peers’, ‘interaction with caregiver’, and ‘verbal communication’ relate to the ASD-defining deficits. Therefore, distinct aspects of ED can be reduced in ASD individuals, which may result in an uneven emotional developmental profile. Disharmonious *cognitive* profiles with specific strengths and weaknesses have been shown in various studies [65]–[68]. Knowledge of these ASD-specific *cognitive* profiles is of value for diagnostic classification and has led to a more comprehensive understanding of the complexity of the disorder. These findings could also apply to the *emotional* developmental profile as measured with the SAED [23]. Therefore, similar to the cognitive profiles, the SAED may be of value for predicting ASD group membership.

Individuals with ASD do not reach the level of functioning that would be expected based on their IQ [69], [70], [71]. Given that the levels of ED and ID are not necessarily paralleled, one possible explanation for the discrepancy between level of functioning and IQ may be delays in ED [51], [72], [73]. The level of ED could be the missing link that - apart from cognitive functions - reduces adaptive functions in ASD. In summary, extrapolating from IQ to the level of ED could be a shortcoming, especially in ASD.

Given the possible impairments and diagnostic value of various aspects of ED on ASD, this study addressed three hypotheses. First (A), the overall level of ED in adults with ID/ASD combined is reduced compared to adults with ID alone. Second (B), additionally ASD is associated with specific deficits in certain domains of ED, e.g., interaction or communication, which results in an uneven profile of ED. Third (C), in the event of there being an overall reduced and uneven profile of ED, this pattern could be of value in predicting ASD group membership.

## Methods

### Ethics Statement

Only existing data from the hospital’s database was used, which was evaluated with written informed consent as part of routine patient care. The SAED and ASD diagnostics comprising SCQ, PDD-MRS, ADOS, and ADI-R were completed by a psychologist (H.K.) or a psychiatrist (SAED only) not involved in the study. All data was anonymized. Evaluation of existing diagnostic data is based on the legal requirements in Berlin (Landeskrankenhausgesetz § 25.1, Version 18.09.2011).

### Setting and Design

This study was conducted at a psychiatric department in a general hospital that includes an in- and outpatient-unit that is specialized for adults with ID in a German metropolitan area. The SAED was applied in a total of *N = *289 adults with ID who were referred to the hospital between 1/2008 and 6/2012. None of the participants lived in a psychiatric long-term institution; all lived with their families or residential homes with a maximum group size of 8 inhabitants. Inclusion criteria were age >18 years, intellectual disability of any degree, hospital referral from 1/2008 to 6/2012, and assessment with the SAED for clinical purposes. To assess ASD group membership, participants were divided into a training sample (1/2008 to 12/2010; *n* = 143) to generate a classification system and a validation sample to verify the newly developed classifier (1/2011 to 6/2012; *n = *146) (cf. data analysis). Subjects initially suffered from a challenging behavior or an additional mental disorder such as schizophrenia or depression, which led to hospital admission. SAED and ASD diagnostics have been evaluated after remission of acute exacerbation of mental illnesses. Data analysis was performed retrospectively from the hospital’s database.

### Participants

In total, *N* = 289 participants (*n* = 103 female, *n* = 186 male) were included in this study. The mean age in the sample was 34.9 years (*SD* = 12.1); the degree of ID was mild (*n* = 78), moderate (*n = *123), and severe-profound (*n* = 88). A total of 102 individuals were diagnosed with ID and ASD, while 187 showed ID only. ASD was already diagnosed *before* referral in 22 individuals (35.5%) diagnosed with ASD after comprehensive assessment (training sample only). Table 2 presents the baseline characteristics including initial mental and neurological disorders and psychotropic drugs of the participants. The single individual could have no, only one, or more than one mental or neurological disorder or medication. Data are shown separately for the training (1/2008–12/2010, *n* = 143) and the validation (1/2011–6/2012, *n* = 146) sample. In the training sample, ASD diagnosis is recognized on admission in *n* = 22 (35.5%) of the individuals who were finally diagnosed with ASD (*n* = 62). In each one out of three individuals with ASD, this diagnosis was known before hospital referral.

**Table 2:** Description of the Study Sample.

	Training Sample (1/2008 to 12/2010)				Validation Sample (1/2011 to 6/2012)			
	Total sample	ID and ASD	ID	*p*	Total sample	ID and ASD	ID	*p*
Number, *n* (%)	143 (100)	62 (43.4)	81 (56.6)		146 (100)	40 (27.4)	106 (72.6)	
Male gender, *n* (%)	96 (67.1)	47 (75.8)	49 (60.5)	.053	80 (54.8)	28 (70.0)	52 (49.1)	.023*
Age in y, Mean (*SD*)	36.06 (12.46)	34.94 (10.31)	36.91 (13.89)	.330^a^	33.75 (11.73)	35.23 (13.09)	33.20 (11.18)	.353^a^
**Severity of ID**, *n*	143	62	81	.018* ^b^	146	40	106	<.001* ^b^
Mild ID, *n* (%)	30 (21.0)	9 (14.5)	21 (25.9)		48 (32.9)	5 (12.5)	43 (40.6)	
Moderate ID, *n* (%)	63 (44.1)	25 (40.3)	38 (46.9)		60 (41.1)	16 (40.0)	44 (41.5)	
Severe-profound ID, *n* (%)	50 (35.0)	28 (45.2)	22 (27.2)		38 (26.0)	19 (47.5)	19 (17.9)	
**Neurological Disorders**, *n*	143	62	81		121	39	82	
Hearing deficits, *n* (%)	8 (5.6)	4 (6.5)	4 (4.9)	.696	9 (7.4)	4 (10.3)	5 (6.1)	.415
Visual deficits, *n* (%)	18 (12.6)	8 (12.9)	10 (12.3)	.921	10 (8.3)	3 (7.7)	7 (8.5)	.875
Movement disorder, *n* (%)	23 (16.1)	4 (6.5)	19 (23.5)	.006*	6 (5.0)	2 (5.1)	4 (4.9)	.953
Seizure disorder, *n* (%)	35 (24.5)	16 (25.8)	19 (23.5)	.746	23 (19.0)	7 (17.9)	16 (19.5)	.838
**Mental Disorders**, *n*	143	62	81		121	39	82	
Dependency disorders (F1x.x), *n* (%)	5 (3.5)	2 (3.2)	3 (3.7)	.877	11 (9.1)	5 (13.2)	6 (7.2)	.292
Schizophrenia (F2x.x), *n* (%)	44 (30.8)	27 (43.5)	17 (21.0)	.004*	24 (19.8)	8 (21.1)	16 (19.3)	.820
Mood disorders (F3x.x), *n* (%)	38 (26.6)	14 (22.6)	24 (29.6)	.344	49 (40.5)	14 (36.8)	35 (42.2)	.580
Neurotic, stress-related and somatoform disorders (F4x.x), *n* (%)	26 (18.2)	12 (19.4)	14 (17.3)	.750	23 (19.0)	8 (21.1)	15 (18.1)	.698
Personality disorders (F6x.x), *n* (%)	16 (11.2)	0 (0)	16 (19.8)	<.001*	7 (5.8)	1 (2.6)	6 (7.2)	.315
**Medication**, *n*	143	62	81		117	38	79	
Antipsychotics- high potency, *n* (%)	74 (51,7)	37 (59.7)	37 (45.7)	.097	56 (47.9)	19 (50)	37 (46.8)	.748
Antipsychotics- low potency, *n* (%)	58 (40.6)	31 (50.0)	27 (33.3)	.044*	30 (25.6)	7 (18.4)	23 (29.1)	.215
Antidepressants, *n* (%)	37 (25.9)	10 (16.1)	27 (33.3)	.020*	25 (21.4)	7 (18.4)	18 (22.8)	.590
Antiepileptic drugs, *n* (%)	52 (36.4)	18 (29.0)	34 (42.0)	.111	30 (25.6)	9 (23.7)	21 (26.6)	.737
Benzodiazepines, *n* (%)	18 (12.6)	10 (16.1)	8 (9.9)	.264	14 (12.0)	8 (21.1)	6 (7.6)	.036*

Differences between ASD and non-ASD individuals were calculated by 2-sided Pearson’s *χ^2^* tests except^ a^ indicates *t*-Test and ^b^ indicates *χ^2^* test for trends;

* indicates *p*<.05.

### Diagnostic Procedures

#### Degree of ID

Participants were grouped as having a mild, moderate, or severe/profound degree of ID. The assignment was based on the results of the ‘Disability Assessment Scale’ (DAS) in most subjects [74]. According to ICD-10, the IQ cut points used were 50 to 70 (mild ID), 35 to 50 (moderate ID), and below 35 (severe to profound ID). In the absence of a current standardized assessment, categorization was made according to the daily living and communication abilities by a physician who was experienced in the field of ID.

#### Diagnosing ASD

Clinical ICD-10 diagnoses for autism and atypical autism (F84.0/F84.1) were based on all of the available information from the current and past medical history, a comprehensive psychiatric and physical examination, a video-based behavior analysis, and standardized psychodiagnostic assessments, such as the ‘Social Communication Questionnaire’ (SCQ) and the ‘Scale for Pervasive Developmental Disorders in Mentally Retarded Persons’ (PDD-MRS) [7], [75]. The SCQ is a categorical scale comprising 40 items that were designed to screen for ASD in children and adolescents (4 to 18 years). The PDD-MRS is a diagnostic interview that is conducted by a trained psychologist with a professional caregiver; it evaluates autistic and problem behaviors of the patient in situations of daily living. This assessment is composed of 12 categorical items, some of which are weighted. Whenever necessary, additionally the ADOS and/or the ADI-R were applied. The ADOS (Autism Diagnostic Observation Schedule) in combination with the ADI-R (Autism Diagnostic Interview-R) are widely used for diagnosing ASD [76]–[78]. The ADOS is a semi-structured observational instrument that assesses social and communicative abilities in individuals with suspicion of ASD [76], [77]. The ADI-R is a semi-structured parental interview that assesses social reciprocity, communication and restrictive, repetitive behaviors as well as interests over a lifetime [78]. Diagnostic classification is assigned by consensus in a multidisciplinary team experienced in ASD and ID, which includes at least one psychiatrist and one psychologist.

#### Assessment of ED

The SAED by Dosen [40], [63], i.e., an interview conducted by a psychologist or psychiatrist trained in developmental psychiatry with a close caregiver, was used to assess ED. The SAED is a semi-structured interview that evaluates the achieved developmental level in 10 basic aspects of ED, in the following domains:

1. Dealing with his/her own body
2. Interaction with a caregiver
3. Experience of self
4. Object permanency
5. Anxiety
6. Interaction with peers
7. Handling of material objects
8. Verbal communication
9. Affect differentiation
10. Aggression regulation

According to the 5 possible developmental levels (cf. Table 1), values from 1 to 5 can be obtained in this ordinal scaled measure corresponding to certain developmental achievements and age-equivalents in typically developing children. Certain statements of observed behavior are typical for the developmental level guide in the interview. Statements in level one (adaptation) in the first domain (dealing with his/her own body) are, for example: 1. Engages with own body (-observing, -playing, -sucking, -masturbating, -picking, -injuring.), 2. Moves in a stereotypical manner (engages in specific movements in the same manner repetitively), and 3. Passive (shows no initiative). The behaviors of a person that most often can be observed mark the developmental level the person is functioning at in a certain domain. Judgment of the developmental level in the 10 different domains listed above results in a ‘profile’ of ED that reflects its various aspects. In this study, the overall judgment of the developmental level was according to the mean level reached over the 10 different domains. Administration of the SAED lasted approximately 20 to 30 minutes. In the training sample (1/2008–12/2010), caregivers were interviewed, while in the validation sample (1/2011–6/2012), the diagnostic interview was conducted with the hospital staff (nurses, special needs caregivers, physicians, pedagogues, therapists, and social workers). The scheme has shown adequate inter-rater reliability (kappa = .75), internal consistency (Cronbach’s alpha = .958) and concurrent validity (r = .512, p<.002) with the Vineland Adaptive Behavior Scales [64].

### Data Analysis

#### (A) Differences in ED in ID individuals with and without ASD

Because the variables of the SAED are ordinal-scaled (level 1 to 5, cf. ‘assessment of ED’), the Mann-Whitney U test was used to assess the differences in the total and domain scores of the SAED between individuals with ID/ASD combined and those with ID alone in the whole sample (*N* = 289). To control for the level of ID, analysis was additionally performed stratified for the level of ID (mild, moderate, severe-profound) and a regression analysis was run to control for the level of ID. Moreover, a Spearman correlation analysis for the levels of ID and ED and these two parameters with ASD was performed. The multiple-testing problem connected with simultaneous testing of the ten items was accounted for by considering a statistic to be significant when passing the Bonferroni threshold *p*<0.05/10 = 0.005.

#### (B) Evenness of the SAED profile

The evenness of a single SAED profile was assessed in three different ways: A SAED profile was defined to be even if at least 6 domains are at the same level *or* at least 4 domains are at the same level and the other domains score at adjacent levels. Second, the difference between the lowest and the highest SAED level within each individual profile was calculated (min-max difference). Finally, the standard deviation was assessed, measuring the variation of the different SAED domains from the average in each subject. A Chi square test and Mann-Whitney U Test were applied to look for differences between ASD and non-ASD individuals, where appropriate.

#### (C) Classification of ASD group membership by SAED analysis

To assess the ability of SAED to predict the ASD group membership, the whole sample (1/2008–6/2012, *N* = 289) was divided into a training sample to generate a classificator for ASD group membership and a validation sample to verify the newly developed classification system in an independent sample. Univariate and bivariate logistic regression were computed to identify items that significantly discriminate between ASD and non-ASD individuals and to correct for the level of ID. A second uni- and bivariate logistic regression analysis assessed the impact of the level of ID and ED on ASD classification.

The items were ranked based on the significance (Mann-Whitney U test), and a partial score was calculated that involved the six highest ranked items. The weights within the new partial score were calculated using a nearest centroid classification that was implemented in the R package cancerclass [79]. Validation of the newly developed SAED algorithm for classifying ASD in ID individuals was conducted by two methods: (1) the multiple random validation strategy as introduced by Michiels et al., and (2) validation in an independent validation sample recruited from 1/2011 to 6/2012 [80]. First, according to the multiple random validation strategy, balanced (equal number of ASD and non-ASD patients) training data sets were randomly drawn from the set of 143 patients. The algorithm was evaluated on test sets that included all of the patients who were not in the training set. For each training set size, 200 random training sets were drawn, and the mean classification rates, including 95% confidence intervals *(CI),* were calculated. Second, all of the patients who were admitted to the hospital between 1/2011 and 6/2012 were included in a second study cohort, the validation sample.

Psychometric properties of the newly developed SAED algorithm were assessed by sensitivity, specificity, likelihood ratio of Cohen’s kappa, and receiver operating characteristics (ROC) (area under the curve, AUC). Missing values were replaced by the mean SAED level. The sensitivities, specificities, and Cohen’s kappa likelihood ratios were calculated using different cut points for ASD: First, analysis was performed for one cut-off ( = 0), which differentiated between ASD and non-ASD group membership, and second for two cut-offs ( = −1; 1), which differentiated between no, possible, and probable ASD. The “possible ASD” group achieved lower values with the ‘SAED for ASD algorithm’ indicating a high sensitivity but lower specificity for ASD group membership. Subjects grouped within the “probable ASD” category gained higher values with the ‘SAED for ASD algorithm’, thus, this category is less sensitive but highly specific for ASD. ROC analyses were computed for the whole sample and separately for different ID groups. According to the recommendations of Cicchetti, Volkmar, Klin, and Showalter (1995), the sensitivity/specificity and the ROC area under the curve (AUC) were evaluated as poor (<0.70), fair (0.70–0.79), good (0.80–0.89), and excellent (0.90–1) [81].

## Results

### (A) Delayed ED in ID and ASD vs. ID Alone

**Figure 2:**
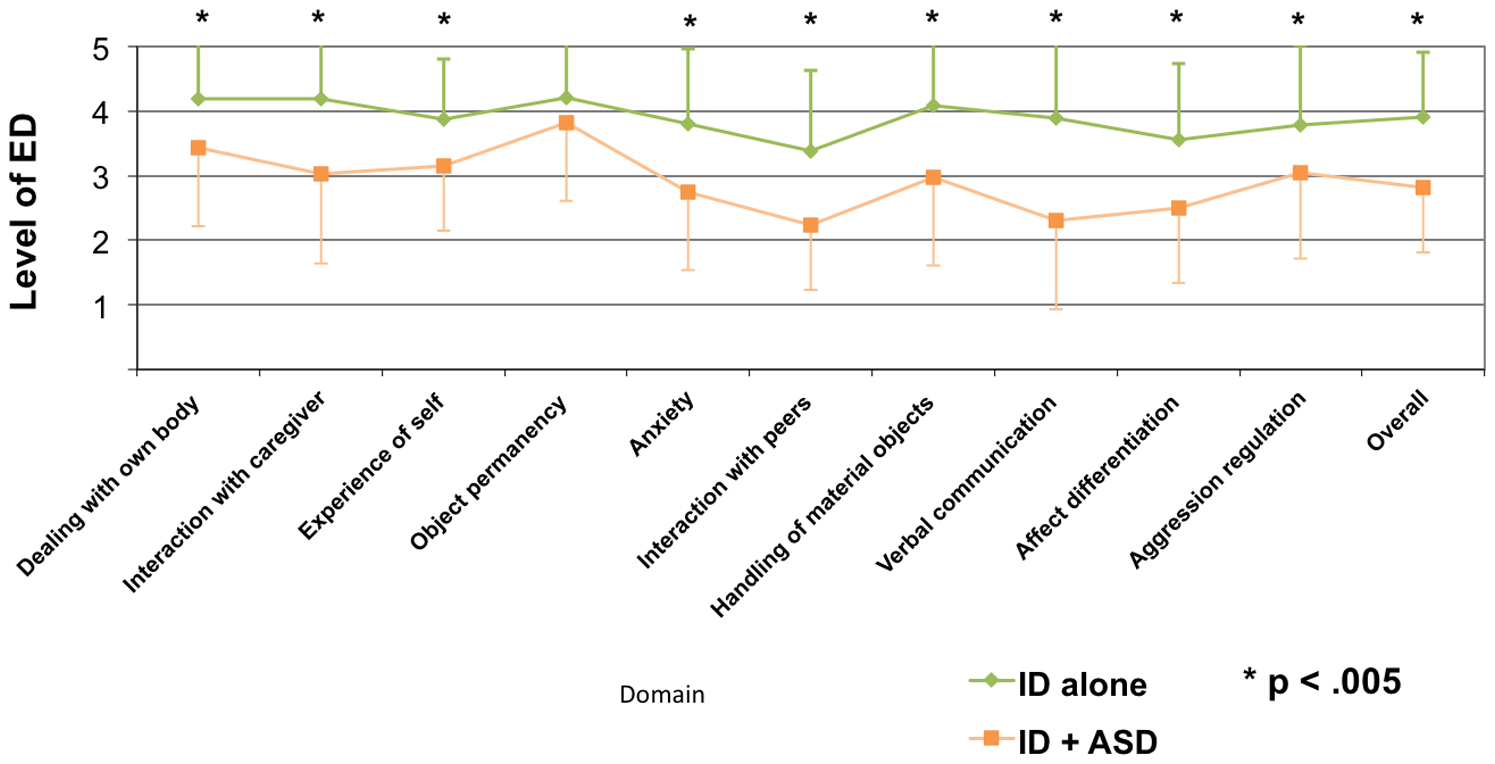
The level of ED in adults with ID/ASD combined and with ID alone. *Note:* Means are marked by dots and standard deviations by whiskers. ID alone = green line; ID and ASD = orange line, * indicates *p*<.005 (Mann Whitney Test).

The level of ED in adults with ID with and without ASD is shown in Figure 2. Adults with ID and ASD showed a lower level of ED compared to adults with ID only. The overall level of ED was 2.81 (*SD* = 1.00) in ASD and ID individuals, which corresponded to the ED of normally developing infants aged 18–36 months (individuation), while those with ID alone achieved an overall developmental level of 3.91 (*SD* = 1.01), which is analogous to 3- to 7-year-old children (identification). These differences were significant as calculated by a Mann-Whitney Test for ordinal-scaled variables with *p*<.0005.

**Figure 3:**
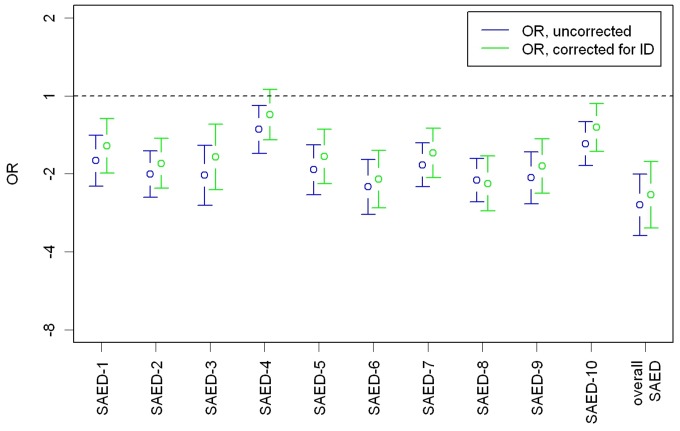
*OR*s of various SAED domains. *Note:* Green dots (means) and whiskers (95% *CI*) indicate correction for the level of ID, black dots and bars indicate values without correction for the level of ID.

Moreover, specific deficits in certain domains of the emotional developmental profile could be observed in individuals with ID and ASD when compared to individuals with ID alone. In fact, differences in the level of ED could be observed in all of the domains except for ‘object permanency’, as seen in Figure 2. The differences are controlled for multiple comparisons by Bonferroni, with *p*<.05/10 = .005. Severity of ID negatively correlated with the level of ED (*r_s_* = -.526, *p*<.001) and both, severity of ID (*r_s_* = .282, *p*<.001) and level of ED (*r_s_* = -.463, *p*<.001), correlated with ASD diagnosis. Severity of ID was coded higher with more severe intellectual impairment, while the level of ED was coded inversely with codes increasing with more advanced levels of ED (c.f. Table 1); therefore the correlation between ID and ED was negative. However, the delays of ED in adults with ID and ASD were independent of the level of ID, as shown by stratified analysis (c.f. Table 3) and regression analysis with odds ratios (*OR*) corrected for ID (c.f. Figure 3).

**Table 3:** SAED in adults with and without ASD - stratified analysis according to the level of ID.

Subsample (n)	Domain of ED^a^										
	1	2	3	4	5	6	7	8	9	10	ED total
**Mild ID (64)**	4.55	4.58	4.30	4.58	4.35	3.78	4.67	4.77	4.05	4.29	4.41
**Mild ID & ASD (14)**	3.79	3.00	3.71	3.71	3.71	2.15	4.29	3.77	3.07	4.15	3.64
***P***	.003*	.264	.011	.002*	.124	.0002*	.453	.010	.010	.634	.024*
**Moderate ID (82)**	4.30	4.34	3.87	4.22	3.71	3.40	4.17	3.99	3.55	3.71	3.94
**Moderate ID & ASD (41)**	3.76	3.24	3.51	4.17	2.85	2.41	3.00	2.63	2.70	3.54	3.02
***P***	.004*	.0005*	.019	.814	.0004*	.0005*	.0005*	.0005*	.0001*	.414	.0005*
**Severe to profound ID (41)**	3.44	3.29	3.20	3.60	2.91	2.73	3.00	2.33	2.83	3.18	3.07
**Severe to profound ID & ASD (47)**	3.06	2.53	2.68	3.54	2.33	2.09	2.55	1.61	2.15	2.27	2.38
***P***	.132	.010	.050	.986	.034	.012	.141	.001*	.002*	.002*	.003*

*p* as calculated differences by Mann-Whitney-U Test. Domain 1–10: * *p*< Bonferroni adjusted alpha (.05/10 = .005); Total score: * *p*<.05.

a Domains of ED: 1. Dealing with own body; 2. Interaction with caregiver; 3. Experience of self; 4. Object permanency; 5. Anxiety; 6. Interaction with peers; 7. Handling with material objects; 8. Verbal communication; 9. Affect differentiation; and 10. Aggression regulation.

Individuals with mild ID (mental age ≈ 9 to 12) showed more advanced levels of ED (*M* SAED score = 4.4) than those with mild ID and additional ASD (*M* SAED score = 3.6). These discrepancies were even more obvious in adults with moderate ID (mental age ≈ 6 to 9 years): The level of ED was 3.9 (which corresponds to 3 to 6-year-old typically developing children) in ID alone, and 3.0 (which corresponds to 1.5 to 3-year-old typically developing children) in ID and ASD. Thus, in individuals with moderate ID, ASD led to intra-individual discrepancies between the mental age (6–9 years) and the emotional age (1.5–3 years), which could not be observed in individuals with ID alone (mental age: 6–9 years; emotional age: 3–6 years). In individuals with severe to profound ID (mental age ≈ 3 years), those with ID alone showed more advanced levels of ED (*M* SAED score = 3.1, which corresponds to 3- to 6-year-old typically developing children) than those with ID and ASD (*M* SAED score = 2.4, which corresponds to 0.5- to 1.5-year-old typically developing infants). Again, within adults with severe to profound ID, intra-individual discrepancies between the mental and emotional age could be observed in ASD but not in non-ASD individuals.

### (B) Uneven ED in Adults with ID Plus ASD vs. ID Alone

Although the profile of ED is straightened out by averaging various individual results, the scatter of the developmental profile in ASD can be seen in Figure 2. The most prominent deficits in the ASD group could be observed in the domains ‘interaction with peer’, ‘interaction with caregivers’, and ‘verbal communication’. Moreover, substantial reductions could also be observed with regard to ‘anxiety’, ‘handling of material objects’, and ‘affect differentiation’. These aspects were less reduced when compared to non-ASD individuals, but SAED domains that remained significantly different were: ‘Dealing with the own body’, ‘experience of self’ and ‘aggression regulation’. No group differences could be observed within the domain ‘object permanence’.

Table 4 demonstrates a significant enhancement of uneven profiles of ED (definition cf. data analysis) in adults with ID and ASD compared to ID alone by the Chi square test.

**Table 4:** Crosstable demonstrating number of even and uneven SAED profiles in the training sample.

Group	Even^a^ *N* (%)	Uneven *N* (%)	Total *N*	Pearson Chi-square (ASD vs. no ASD)
ID & ASD	43 (42.2)	59 (57.8)	102	
ID	120 (64.2)	67 (35.8)	187	*p*<0.0005

a Even profile: at least 6 domains are at the same emotional level *or* at least 4 domains are at the same level, and the other domains score at adjacent levels.

Moreover, intra-individual variability of the SAED profile was assessed by calculating the difference between the lowest and the highest developmental level that was achieved (the min-max difference). While adults with ID and additional ASD (n = 102) showed an average min-max difference of 2.79 (*SD = *1.06) levels, this difference was significantly smaller in adults with ID only *(n* = 187; *M* = 2.10; *SD* = 1.04), as calculated by the Mann-Whitney Test (*p*<.0005).

Finally, variations within a single SAED profile were also assessed by standard deviation, which was greater in ID/ASD combined (*M* = .977; *SD* = 0.33) compared to ID only (*M* = 0.75; *SD* = 0.33). This difference was significant as indicated by the Mann-Whitney Test (*p*<.0005).

### (C) Classification of ASD Group Membership Using the SAED

Items were ranked based on the significance of the separation between individuals with and without ASD. A partial score was calculated that involved the items best discriminating ASD from non-ASD individuals indicating ASD group membership: SAED Score for ASD = 0.299 SAED-overall +0.408 SAED-8+0.316 SAED-9+0.304 SAED-7+0.291 SAED-6+0.249 SAED-5–6.30.

#### Psychometric properties of the SAED algorithm

**Figure 4:**
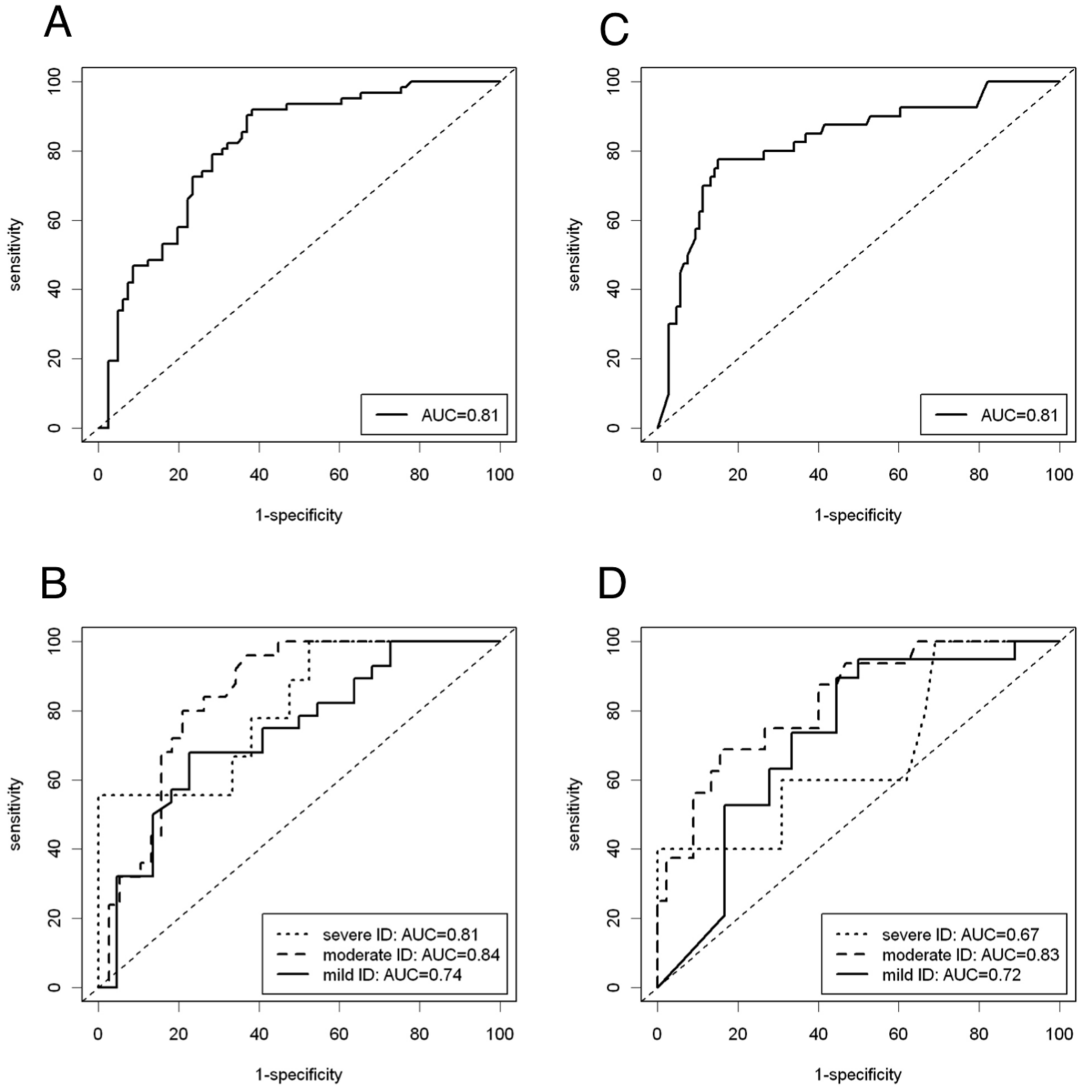
ROC obtained with the tentative SAED algorithm indicating ASD group membership. **A) Whole training sample.** *Note:* ROC curve = solid line, reference line = straight broken line. **B) Stratified analysis, training sample.** *Note:* mild ID = solid line, moderate ID = broken line, severe-profound ID = dotted line, reference line = straight broken line. **C) Whole validation sample.** *Note:* ROC curve = solid line, reference line = straight broken line. **D) Stratified analysis, validation sample.** *Note:* mild ID = solid line, moderate ID = broken line, severe-profound ID = dotted line, reference line = straight broken line.

Using this newly developed algorithm, the ROC and the AUC was assessed for the training sample, including all levels of ID (c.f. Figure 4A) and controlled for the influence of ID by a stratified analysis (cf. Figure 4B). Diagnostic validity analysis was performed for one cut-off ( = 0), which differentiated between ASD and non-ASD, and for two cut-offs ( = −1; 1), which differentiated between no, possible, and probable ASD. Sensitivity, specificity, and Cohen’s kappa were assessed as shown in Table 5 for the different cut-offs.

**Table 5:** Psychometric properties of the newly developed SAED algorithm for classifying ASD.

Sample	Sensitivity	Specificity	*p*
**Cut off = 0 (ASD/no ASD)**			
Training sample	77.4	71.6	<.0005
Validation sample	82.5	64.2	<.0005
**Cut off = 1 (probable ASD)**			
Training sample	53.2	82.7	<.0005
Validation sample	77.5	76.4	<.0005
**Cut off = −1 (possible ASD)**			
Training sample	91.9	60.5	<.0005
Validation sample	87.0	51.9	<.0005

*p* calculated by a 2-sided Fisher Test.

#### Multiple random validation strategy

**Figure 5:**
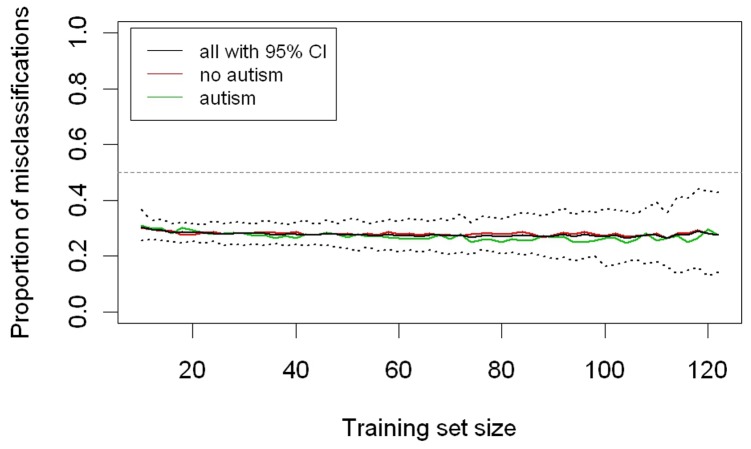
Multiple random validation strategy. *Note:* black line = proportion of misclassification; dotted line = 95% *CI*; the 95% *CI* below the broken straight 50% line indicates significance. The accuracy of the tentative SAED algorithm remains the same across different sample sizes.

A multiple random validation strategy revealed a proportion of misclassifications of approximately 25% (cf. Figure 5). Conversely, 75% of the patients were classified correctly using the tentative SAED algorithm, as indicated by this statistical model. Classification success was found to be independent within a large range of subjects, which suggested that the sample size of the present cohort is large enough to predict group membership.

#### Validation sample

Psychometric properties of the SAED algorithm developed in the training sample (1/2008 to 12/2010, *n* = 143) were assessed in a second, independent study cohort that was recruited from 1/2011 to 6/2012, *n* = 146. Again, a standardized ASD assessment was applied to identify all of the ASD individuals within this sample. The tentative SAED algorithm for classifying the ASD group membership suggested above was administered, and psychometric properties were evaluated. The ROC Curve for the validation sample, which included all ID levels, is shown in Figure 4C, and the stratified analysis according to the different ID levels is shown in Figure 4D. In accordance with the results from the multiple random validation strategy in the training sample, the AUC was.81. In the stratified analysis, the AUC levels varied from.67 (severe to profound ID) to.83 (moderate ID). The sensitivity and specificity for either cut-off (−1/1; 0) can be seen in Table 5.

#### Contribution of level of ID and ED on ASD diagnosis

An univariate regression analysis revealed a predictive value for both, severity of ID (*OR* = 2.29, *p*<.001, 95% *CI* [1.16, 3.25], *R^2^* = 0.11) and level of ED (*OR* = 0.37, *p*<.001, 95% *CI* [0.28, 0.48], *R^2^* = 0.28) for the ASD classification. In a bivariate regression model (*R^2^* = 0.29), however, only the level of ED was a significant predictor for ASD (*OR* = 0.39, *p*<.001, 95% *CI* [0.29, 0.53]), while severity of ID did no longer reach a significant effect (*OR* = 1.21, *p* = .374, 95% *CI* [0.80, 1.83]). This indicates a 2.7 fold risk for ASD with every decreasing level of ED.

## Discussion

Given that ASD is a disorder of development, certain levels of function achieved in the development of emotion processing, such as attachment and self-regulation, can be affected as well. This study addressed three main issues: The first issue (A) was whether adults with ID and ASD show a reduction, i.e., a delay in overall ED compared to adults with ID only. Second (B), differences of the profile over various domains of ED in ASD vs. non-ASD were evaluated. Third (C), the impact of the overall level and the distinct profile of ED were assessed for predicting the ASD group membership.

The main finding of the study was an (A) overall lower and (B) more uneven profile of ED in adults with ID and ASD compared to those with ID alone. This reduction and specific pattern was independent of the level of ID as shown by stratified and regression analysis. (C) The observed ASD-specific deficits could be helpful in classifying ASD group membership in adults with ID.

Within the training sample (1/2008–12/2010), at the time of hospital referral, ASD had been diagnosed in 22 patients, while after standardized ASD assessment, finally, *n* = 62 were diagnosed with ASD. Thus, before admission, ASD diagnosis was known in only 22 out of the 62 (35.5%) who were finally diagnosed with ASD. In an Italian ID cohort recruited from a residential home, La Malfa et al. found even fewer ASD cases (20%) being diagnosed before a systematic ASD assessment [5]. Similar results were found in a population-based study in South Korea, with 2/3 being undiagnosed [2]. These numbers highlight that in the ID and in the general population, there is a very large number of people in the autism spectrum who are undiagnosed and, as a result, are untreated.

### (A) Overall ED Group Differences

The overall level of ED was lower in adults with ID and ASD compared to adults with ID alone, as assessed by the Mann-Whitney test. A Chi square test for trends revealed higher levels of ID in the ASD/ID sample compared to the controls with ID only. To control for this potential bias, differences of the level of ED in ASD and non-ASD individuals were assessed in subsamples stratified for the level of ID (cf. table 3). Moreover, a regression analysis was run controlling the level of ID (cf. Figure 3). The effect of lower levels of ED in the ASD/ID combined sample versus the ID only sample was stable in both analyses controlled for ID. This is further supported by higher correlation coefficients of the level of ED than of ID with ASD diagnosis. Although the overall level of ED has not yet been evaluated in the ASD population to date, different studies that assess its related aspects as outlined in Figure 1 support this finding. Totsika et al. (2011) found the highest levels of emotional problems in ASD children [82]. Moreover, adaptive skills that result from the overall level of ED (cf. introduction, Figure 1) are highly correlated [64]. Accordingly, Matson et al. found a strong impact of additional ASD on adaptive skills as measured by the Vineland Adaptive Behavior Scale in ID individuals [83]. Similar results were obtained in higher functioning individuals [84]. In another study, however, solely basic social skills as measured by one of the subdomains of the Vinland Adaptive Behavior Scales did not contribute to differentiate between ASD and non-ASD in children with mild and moderate ID [85]. Yet, within the same study, more subtle social skills assessed with first, the Children’s Social Behaviour Questionnaire, and second, the Communication domain of the Vinland Adaptive Behavior Scale, were conducive to ASD diagnosis. Finally, distinct delays in sensorimotor development that share certain aspects with overall ED (cf. Figure 1) have been described in various studies in children with ASD [86]–[88]. In summary, different studies that evaluated social, sensorimotor, and communicative development, which in part overlap with the overall ED (cf. Figure 1), and adaptive skills, which result from the level of ED, support our finding of a reduction in ED in ID individuals with additional ASD compared to those with ID only. Hence, besides the symptom-defining ASD core deficits, the overall level of ED is affected as well. To understand a given behavior, adjust expectations accordingly, and fulfill the emotional needs of an individual with developmental disabilities, knowledge of the level of ED is pivotal and cannot be deduced from the level of ID [89], [90], [91], [92].

### (B) Differences in Certain Domains of ED

Aside from the overall low level of ED in adults with ID and ASD, specific deficits can be observed in certain domains. The clear developmental deficits in the domains 8 ‘verbal communication’, 6 ‘interaction with peers’, and 2 ‘interaction with caregiver’ are consistent with the diagnostic criteria of ASD. This finding further supports the validity of the SAED.

Moreover, deficits in self-representation domains such as ‘experience of self’ and ‘dealing with own body’ were reported in ASD individuals. The delays in ‘experience of self’ were independent of the severity of the ID and were strongly associated with the presence of ASD. According to Hobson, various aspects of the developing self are important prerequisites for communication and thinking and could be pivotal for the understanding of ASD [93]. Aside from these cognitive aspects about the self, delays also were observed in the domain ‘dealing with the own body’. Altered body motion perception and motor abilities are commonly reported in individuals with ASD [94]–[96] and could result in mental disorders such as depression or dysmorphic disorder [97].

Developmental delays could also be observed in the emotion systems, such as ‘affect differentiation’, ‘anxiety’, and ‘aggression regulation’. Individuals with ASD without ID appear to be relatively competent in dealing with basic emotions, such as joy and sadness, but have more difficulty with complex emotions such as pride and shame [98], whereas in individuals with ASD and ID, emotional difficulties are more widespread [99]. These difficulties could be partly explained by the delays in ‘affect differentiation’ that were observed in this study. Interestingly, an emotion training program in children with ASD and ID showed limited efficacy in teaching basic emotion recognition skills [100]. On the background of our study results, initiation of a maturing process of ED could be an alternate approach to lay the developmental foundations for emotion recognition and regulation in a population with learning difficulties [101]–[103]. Improvement of emotion recognition skills is of particular importance because reduced emotional knowledge is associated with enhanced aggression and anger, reduced school success and prosocial behavior [104], [105].

Our study showed a delayed level in the ‘anxiety’ domain in ASD individuals compared to non-ASD individuals. According to the developmental approach, causes for anxiety change over the different developmental levels, i.e., anxiety due to intense or unknown stimuli (level 1), separation anxiety (level 2), anxiety due to loss of autonomy (level 3), fear of failure (level 4) and social anxiety, i.e., not being accepted (level 5). A broad body of literature describes an enhanced occurrence of anxiety disorders in ASD individuals (for a review, see: Howlin, 2004) [106]. This increased susceptibility for anxiety disorders could in part be caused by the observed developmental delays, for example, the type of anxiety that is related to the level of emotional development [63]. Thus, recognition of basic emotional needs associated with a certain level of ED could further prevent mental health problems from emerging.

According to this study results, ASD was associated with developmental delays in ‘aggression regulation’. An increase in aggression, especially in self-injurious behavior, is known in ASD individuals [106], [107]. The analysis of causes of aggressive behavior is complex. In individuals with ID and ASD, besides physical disorders, functional analysis of aggressive behavior and alterations in sensory perceptions are the most common approaches in the understanding and treatment of aggression. The developmental approach could further help to understand this dysfunctional behavior because certain developmental levels are associated with distinct processes of self-organization and action systems [31]. From the developmental viewpoint, aggression in SAED level 1 is often due to discomfort/pain or auto-stimulation; aggression in level 2 arises because of insecure attachment and separation anxiety, and in level 3 is from autonomic efforts [108], [109].

In addition to impairments in contact with other people and the self, delays in ‘handling of material objects’ can be observed in our study. These deficits could lead to the previously described impairments in executive functions in ASD [10], [15]. Thus, belonging to the pervasive developmental disorders, ASD not only affects contact with the social world but also affects the relation to the self and to material objects.

In the whole sample and across the different levels of ID, no differences in the domain ‘object permanence/separation anxiety’ can be observed. The development of an inner representation of the outside world as revealed by object permanence is a fundamental prerequisite for secure bounding and therefore must be assessed when evaluating the overall ED. Not being impaired with regard to ‘object permanence’, adults with ID and ASD can form an inner picture of the surrounding environment and keep this picture in their mind. Interestingly, Bernabei et al. found already in 3-year-old children with ASD an uneven developmental profile with object permanence being the most advanced skill [86]. This specific strength in the developmental profile of ASD individuals is supported by various other studies [87], [108], [110].

### (C) ASD Group Membership

A number of studies have shown a disharmonious profile of intelligence in ASD individuals with and without ID at different ages [65]–[68]. These specific cognitive patterns are to be considered as a possible diagnostic aid [111]. In addition to these distinctive *cognitive* profiles, according to our results, also the *emotional* profile was uneven in adults with ASD when compared to adults with ID only. This scatter in ASD individuals was observed by a Chi square test, the magnitude of the min-max differences, and the standard deviation within a single SAED profile. An inhomogeneous developmental profile could already be observed at very early ages [86], [112]–[114]. Our results extend these findings into adulthood. However, longitudinal studies are necessary, to evaluate this hypothesis. Further research is needed to analyze the influence of these uneven emotional developmental profiles on the rates and severity of challenging behavior or psychiatric diseases. Taken together, adults with ID and ASD depict an uneven level of ED with distinct strengths and deficits. Thus, a certain pattern of emotional development can be identified, which could serve as an aid in the process of diagnosing ASD in individuals who have severe intellectual and multiple disabilities. Independent from the severity of ID, there is a 2.7 fold increase for ASD with every decreasing level of ED.

Because of the distinct pattern of the SAED in people with ID, we evaluated its impact for classifying ASD group membership in our cohort. For this purpose, the whole sample was divided into a training sample (recruited from 2008 to 2010) and a validation sample (recruited from 2011 to 6/2012). By item ranking on the basis of significance and using a partial SAED score, with the training sample, an algorithm was generated that lead to promising results with regard to ASD classification. Multiple random validation strategies applied to the training sample was supportive for assignment of the newly developed tentative algorithm in an independent sample. Indeed, the impact of the SAED criteria for ASD classification in adults with ID was evident in the validation sample, as shown by psychometric properties, the ROC curve, and the AUC of.81, which is ‘good’ according to the recommendations of Cicchetti et al. [81]. Thus, the SAED algorithm can be recommended as a diagnostic aid for ASD in the ID population. However, this approach is not a tool that is specifically designed for this purpose, and it is not oriented at the ASD diagnostic criteria. The SAED is not expected to substitute for a specific ASD measure; instead, it can add a further perspective, especially in individuals with profound or multiple disabilities. Similarly, a cognitive profile has been proposed to apply in ASD diagnostics [111]. By using the SAED algorithm for judgment of ASD group membership *in addition to* the DSM-IV-TR/ICD-10 based instruments, such as the ADOS and the ADI-R, etiological factors are incorporated in the process of differential diagnosis and classification. Thereby, solely descriptive and categorical models of psychopathology can be adapted for individuals with developmental disorders, as claimed by various researchers [31], [115]. Considering the overlap of certain aspects of the SAED and ASD such as the communication and interaction domains, one may argue not to measure the level of ED but ASD defining symptomatology with the SAED. However, delays associated with ASD could also be observed in SAED domains such as ‘dealing with own body’, ‘handling with material objects’, and ‘aggression regulation’. These aspects of the SAED cannot directly be related to ASD symptoms. Moreover, even within the communication and interaction domains of the SAED, the level of ED is deduced from the developmental perspective and not according to ASD associated competencies. Furthermore, not all individuals with ASD also show delays in ED. Given that ASD is a pervasive developmental disorder affection of ED is not surprising. Despite distinct overlaps with ASD, the ‘concept of ED’ as assessed with the SAED is going beyond sole ASD defining symptomatology. It may add value to a comprehensive understanding of the disorder and its associated behavioral problems.

This study has a number of limitations that must be considered when interpreting the results. First, the SAED must be normed in a normative population, and its factor structure must be assessed. However, reliability and validity data exist from a study described in detail in the methods section [64]. Second, standardized cognitive testing is available only in selected cases. In most subjects, the level of ID was assessed by the disability assessment scale (DAS), which indicates the degree of ID [74]. The DAS and the SAED provide only rough estimates of the cognitive and emotional developmental level. Third, in the training sample (1/2008–12/2010), the SAED was assessed by interviewing close caregivers or family members, while in the validation study, the SAED was assessed within a multidisciplinary team in the hospital. Overall, the ratings within the hospital team showed lower levels of ED when compared to the caregiver interview. Albeit overall similar predictive abilities of the tentative SAED algorithm, this approach led to higher sensitivity and lower specificity results in the validation sample. Finally, the analysis was performed in a clinical setting comprising a study sample with high incidences of various mental and neurological disorders as described in detail in the method section. These additional disorders and the therewith-associated medication may confound the study results. To reduce this effect, ASD was diagnosed and level of ED and ID was evaluated after remission of acute exacerbation of mental disorders. Moreover, this variable applied to both, cases and controls.

In conclusion, the level of ED was reduced and uneven in adults with ID and ASD compared to adults with ID only. The distinct developmental profile in ASD individuals could serve as a diagnostic aid when evaluating ASD in adults with intellectual and possibly multiple disabilities. Because maladaptive behavior can be understood as an expression of unmet needs due to discrepancies between the emotional and the mental ages or within the developmental profile, accounting for these discrepancies could become a vantage point for additional treatment options.
